# Intratumoral vidutolimod as monotherapy or in combination with pembrolizumab in patients with programmed cell death 1 blockade–resistant melanoma: Final analysis from a phase 1b study

**DOI:** 10.1002/cncr.70022

**Published:** 2025-08-03

**Authors:** Mohammed M. Milhem, Yousef Zakharia, Diwakar Davar, Elizabeth I. Buchbinder, Theresa Medina, Adil Daud, Antoni Ribas, Bartosz Chmielowski, Jiaxin Niu, Geoffrey T. Gibney, Kim Margolin, Anthony J. Olszanski, Inderjit Mehmi, Takami Sato, Montaser Shaheen, Luping Zhao, Heather Kelley, Hong Liu, Sujatha Kumar, Dmitri Bobilev, Arthur M. Krieg, James E. Wooldridge, John M. Kirkwood

**Affiliations:** ^1^ University of Iowa Iowa City Iowa USA; ^2^ University of Pittsburgh Medical Center Pittsburgh Pennsylvania USA; ^3^ Dana‐Farber Cancer Institute Boston Massachusetts USA; ^4^ University of Colorado Denver Aurora Colorado USA; ^5^ University of California San Francisco San Francisco California USA; ^6^ Jonsson Comprehensive Cancer Center University of California Los Angeles Los Angeles California USA; ^7^ Banner MD Anderson Cancer Center Gilbert Arizona USA; ^8^ Lombardi Comprehensive Cancer Center MedStar Georgetown University Hospital Washington DC USA; ^9^ Saint John's Cancer Institute Santa Monica California USA; ^10^ Fox Chase Cancer Center Philadelphia Pennsylvania USA; ^11^ The Angeles Clinic and Research Institute Cedars‐Sinai Affiliate Los Angeles California USA; ^12^ Thomas Jefferson University Philadelphia Pennsylvania USA; ^13^ The University of Texas Health Science Center at San Antonio San Antonio Texas USA; ^14^ Checkmate Pharmaceuticals Cambridge Massachusetts USA; ^15^ Fusion Pharmaceuticals Boston Massachusetts USA; ^16^ Zola Therapeutics Needham Massachusetts USA; ^17^ Hillman Cancer Center University of Pittsburgh Medical Center University of Pittsburgh Pittsburgh Pennsylvania USA

**Keywords:** CMP‐001, immunotherapy, melanoma, pembrolizumab, phase 1b, Toll‐like receptor 9 (TLR9) agonist, vidutolimod

## Abstract

**Background:**

New treatment options are needed for patients with metastatic anti–programmed cell death 1 (PD‐1)–resistant melanoma. The final analysis of a phase 1b study evaluating the Toll‐like receptor 9 agonist vidutolimod is reported here.

**Methods:**

This two‐part, open‐label, multicenter, phase 1b study in adults with metastatic/unresectable anti–PD‐1–resistant melanoma evaluated the safety and clinical activity of intratumoral vidutolimod plus systemic pembrolizumab (part 1) or vidutolimod alone (part 2). Two vidutolimod formulations were evaluated with different concentrations of polysorbate (PS20‐A, 0.005%–0.01% polysorbate 20; PS20‐B, 0.00167% polysorbate 20). Key end points were safety and investigator‐assessed objective response rate (ORR; Response Evaluation Criteria in Solid Tumors, version 1.1).

**Results:**

A total of 159 patients were treated in part 1 (PS20‐A, *n* = 98; PS20‐B, *n* = 61), and 40 patients were treated in part 2. Any‐grade treatment‐emergent adverse events (TEAEs) occurred in 100.0% of patients. Grade ≥3 TEAEs occurred in 55.3% (part 1) and 37.5% (part 2) of patients. No treatment‐related deaths occurred. Best ORR was 23.5% (95% CI, 15.5%–33.1%; complete response [CR], 7.1%) for vidutolimod PS20‐A plus pembrolizumab, 11.5% (95% CI, 4.7%–22.2%; CR, 1.6%) for vidutolimod PS20‐B plus pembrolizumab, and 20.0% (95% CI, 9.1%–35.6%) for vidutolimod monotherapy. Median duration of response was 25.2 months with vidutolimod PS20‐A plus pembrolizumab, 11.4 months with vidutolimod PS20‐B plus pembrolizumab, and 5.6 months with vidutolimod monotherapy.

**Conclusions:**

Vidutolimod PS20‐A alone or in combination with pembrolizumab had an acceptable safety profile and promising clinical activity in patients with PD‐1 blockade–resistant melanoma.

## Abstract 2

Vidutolimod is a virus‐like particle containing a CpG‐A Toll‐like receptor 9 agonist. Vidutolimod alone or in combination with pembrolizumab had an acceptable safety profile, and showed antitumor activity in patients with programmed cell death 1 blockade–resistant melanoma.

## INTRODUCTION

Nonresponse to programmed cell death 1 (PD‐1) blockade is associated with a lack of preexisting IFN‐γ–secreting CD8+ T cells in the tumor microenvironment (TME), which suggests that impaired T‐cell priming or trafficking to tumor tissues might be involved in immune resistance.^1^, ^2^, ^3^ Toll‐like receptor 9 (TLR9)–mediated activation of intratumoral plasmacytoid dendritic cells (pDCs) induces type I interferon secretion, which is essential for inducing antitumor immunity, and may improve priming and/or homing of T cells.^4^, ^5^

Vidutolimod, a first‐in‐class TLR9 agonist, bridges innate and adaptive immunity by activating pDCs. Vidutolimod is a CpG‐A oligodeoxynucleotide packaged within a noninfectious, immunogenic virus‐like particle (VLP) that induces anti‐VLP antibody production, which facilitates pDC activation via the costimulation of TLR9 and Fc receptors,^6^, ^7^, ^8^ which results in IFN‐α secretion.^9^, ^10^, ^11^, ^12^ Intratumoral delivery of VLP‐encapsulated CpG‐A enables a specific antitumor response by selectively activating tumor‐associated pDCs and conventional DCs that present tumor antigens to CD8+ T cells,^13^ and culminates in antitumor CD8+ T‐cell activation with improved systemic antitumor activity.^8^, ^14^

TLR9‐induced T cells express PD‐1, which inhibits their function.^15^ Hence, combining intratumoral vidutolimod with systemic anti–PD‐1 may potentially improve response to TLR9 agonists^14^ and overcome resistance to single‐agent PD‐1 blockade by triggering an interferon response to induce and attract antitumor T cells, which converts non–T cell–inflamed TMEs into an inflamed state. We previously reported part 1 dose‐escalation results of this study, with an objective response rate (ORR) of 25% (11 of 44) in patients with advanced melanoma receiving vidutolimod plus pembrolizumab.^16^ The study continued to enroll patients into the part 1 dose expansion, and added a part 2 cohort receiving vidutolimod monotherapy. Here, we report the final analysis of this study evaluating intratumoral vidutolimod with or without systemic pembrolizumab in patients with PD‐1 blockade–resistant melanoma.

## MATERIALS AND METHODS

### Patients

This phase 1b study enrolled patients with histologically confirmed metastatic/unresectable cutaneous melanoma, Eastern Cooperative Oncology Group performance status of 0 or 1, and measurable disease per Response Evaluation Criteria in Solid Tumors, version 1.1 (RECIST v1.1). Patient eligibility required ≥1 lesion with a longest diameter of ≥0.5 cm amenable to intralesional injection. Inclusive of all protocol amendments, eligible patients were required to have either progressive disease (PD) per RECIST v1.1, be on current or prior treatment with any Programmed Death Ligand 1 (PD‐(L)1) inhibitors administered alone or in combination, or have stable disease (SD) per RECIST v1.1 after ≥12 weeks of anti–PD‐(L)1 therapy. There was no maximum time limit since last anti–PD‐(L)1 treatment. Patients with brain metastases were excluded unless the lesions were previously treated and clinically stable. Section SA1 provides additional information on eligibility criteria.

### Study design

Part 1 of this open‐label, multicenter, nonrandomized, phase 1b study (ClinicalTrials.gov identifier NCT02680184) included dose escalation and dose expansion of intratumoral vidutolimod plus intravenous (iv) pembrolizumab. Dose escalation followed a standard 3 + 3 design, and enrolled 44 patients at dose levels from 1 to 10 mg of vidutolimod. Part 1 dose expansion and part 2 (vidutolimod monotherapy) enrolled patients at either 5 or 10 mg until protocol amendment 7, which established 10 mg as the dose for all newly enrolled patients and allowed ongoing patients the option to receive 10 mg of vidutolimod. The first patient in the part 1 dose‐escalation and dose‐expansion study was enrolled on April 14, 2016. In part 2, the first patient was enrolled on July 25, 2017.

Two vidutolimod formulations were evaluated with different concentrations of polysorbate (PS20‐A [0.005%–0.01% polysorbate 20] and PS20‐B [0.00167% polysorbate 20]). For PS20‐B, the injection volume was increased by diluting vidutolimod with normal saline in a 1:6 ratio in order to inject multiple lesions in patients with increased tumor burden. However, subsequent analytical studies demonstrated that the concentration of polysorbate 20 in PS20‐B was below the critical micelle concentration,^17^ which reduced the bioavailability of vidutolimod and in vivo antitumor activity in a murine tumor model (data on file). Additionally, patients treated with saline‐diluted vidutolimod PS20‐B had decreased antitumor activity. Therefore, vidutolimod PS20‐A was selected for further clinical development.

The objective of the part 1 dose‐expansion phase of this study was to characterize safety and obtain preliminary evidence of antitumor activity of vidutolimod plus pembrolizumab. Part 2 evaluated the safety and efficacy of vidutolimod when administered as monotherapy.

The study protocol and its amendments were approved by the institutional review boards of all participating institutions (Table S1), and the study was conducted in accordance with the Declaration of Helsinki and the International Conference on Harmonisation Guidelines for Good Clinical Practice. All patients provided voluntary written informed consent.

Vidutolimod (1, 3, 5, 7.5, or 10 mg) was injected intratumorally into ≥1 lesion weekly for 7 weeks, followed by every 3 weeks (Q3W) thereafter (schedule A), or else weekly for 2 weeks, followed by Q3W thereafter (schedule B). Section SA2 provides guidance on vidutolimod injection and tumor selection. Premedication(s) (i.e., iv fluids, antipyretics, and antiemetics) and a postdosing 4‐h observation period through the first 6 weekly vidutolimod doses were recommended on the basis of early safety data. A subsequent protocol amendment recommended treatment with stress dose corticosteroids before or immediately after injection of vidutolimod in patients with adrenal insufficiency because of an increased risk of moderate‐to‐severe adverse events (AEs) such as hypotension. Pembrolizumab was administered intravenously according to the prescribing information.^18^ Study treatment continued until unacceptable toxicity, PD, investigator decision, or withdrawal of consent. Treatment could be continued beyond PD at the discretion of the investigator.

### Clinical end points

Safety assessments were performed at baseline and at each vidutolimod dosing visit. All AEs reported were considered treatment emergent, and the relationship of AEs to study treatment was determined by the investigator.

Tumor assessments were conducted at screening, every 12 weeks during treatment, and at the end‐of‐treatment visit. Antitumor activity was assessed via best ORR (confirmed/unconfirmed), time to response, duration of response (DOR), progression‐free survival (PFS) by investigator assessment per RECIST v1.1, and overall survival (OS). Tumor regression in patients who continued study treatment after PD per RECIST v1.1 was assessed by the investigator according to immune‐based RECIST (continuation of treatment postprogression was permitted at the discretion of the investigator).^19^

### Statistical analysis

The intention‐to‐treat population included all patients who received ≥1 dose of vidutolimod. Descriptive statistics included means with standard deviations or medians with ranges for continuous variables and counts and percentages for categorical variables; 95% CIs of ORR were calculated via exact methods (Clopper–Pearson). Patients without a postbaseline follow‐up tumor assessment were considered nonresponders. Median DOR, PFS, and OS were estimated via the Kaplan–Meier method. This article includes data from planned analyses (per the statistical analysis plan) and ad hoc analyses. Analyses were performed with SAS software, version 9.4 (SAS Institute, Cary, North Carolina).

## RESULTS

### Patient population

Between April 14, 2016, and April 26, 2021, 159 patients, including 44 patients from the dose‐escalation part of the study, were enrolled in part 1 (vidutolimod plus pembrolizumab), and 40 patients were enrolled in part 2 (vidutolimod monotherapy). In part 1, 98 patients received vidutolimod formulation PS20‐A, and 61 patients received PS20‐B. In part 2, 31 patients began treatment with vidutolimod PS20‐A, and nine patients began treatment with PS20‐B. Patient demographics and baseline characteristics are shown in Table 1, and were similar between patients receiving vidutolimod plus pembrolizumab or vidutolimod monotherapy. In part 1 (formulation PS20‐A), 4.1%, 11.2%, 34.7%, and 41.8% of patients had complete response (CR), partial response (PR), SD, and PD, respectively, as their best response to prior anti–PD‐1 therapy. In part 2, 7.5%, 10.0%, 45.0%, and 22.5% of patients had CR, PR, SD, and PD, respectively, as their best response to prior anti–PD‐1 therapy.

**TABLE 1:** Patient demographics and clinical characteristics.

	Part 1: Vidutolimod + pembrolizumab (dose escalation and expansion)		Part 2: Vidutolimod monotherapy
	Vidutolimod PS20‐A + pembrolizumab (*N* = 98)	Vidutolimod PS20‐B + pembrolizumab (*N* = 61)	Vidutolimod^a^ (*N* = 40)
Age, median (range), years	63 (31–86)	65 (30–90)	68 (30–89)
Male sex, No. (%)	46 (46.9)	43 (70.5)	26 (65.0)
Race, No. (%)			
White	94 (95.9)	59 (96.7)	38 (95.0)
Black or African American	0	1 (1.6)	2 (5.0)
Asian	1 (1.0)	0	0
Other	2 (2.0)	1 (1.6)	0
ECOG PS, No. (%)			
0	67 (68.4)	37 (60.7)	20 (50.0)
1	31 (31.6)	24 (39.3)	20 (50.0)
*BRAF* V600E positive, No. (%)	34 (34.7)	24 (39.3)	12 (30.0)
Received prior BRAF/MEK inhibitor	11 (11.2)	10 (16.4)	7 (17.5)
LDH levels, No. (%)			
Normal/low	57 (58.1)	32 (52.5)	22 (55.0)
High	38 (38.8)	29 (47.5)	18 (45.0)
Unknown	3 (3.1)	0	0
Disease location, No. (%)			
Skin only	6 (6.1)	6 (9.8)	24 (60.0)
Lymph nodes ± skin	22 (22.4)	9 (14.8)	26 (65.0)
Soft tissues ± skin and lymph nodes	14 (14.3)	7 (11.5)	14 (35.0)
Bone metastases without visceral metastases	4 (4.1)	3 (4.9)	2 (5.0)
Any visceral metastases	52 (53.1)	36 (59.0)	16 (40.0)
Other visceral metastases without brain or liver metastases	29 (29.6)	19 (31.1)	1 (2.5)
Any liver metastases	20 (20.4)	13 (21.3)	0
Any liver or brain metastases	3 (3.1)	4 (6.6)	1 (2.5)
Lung metastases without other visceral metastases	0	0	0
Prior therapies received, median (range)	2 (1–8)	2 (1–6)	2 (1–7)
1, No. (%)	39 (39.8)	26 (42.6)	17 (42.5)
2 or 3, No. (%)	39 (39.8)	25 (41.0)	14 (35.0)
≥4, No. (%)	20 (20.4)	10 (16.4)	9 (22.5)
Prior checkpoint inhibitor therapies received, No. (%)	98 (100)	61 (100)	40 (100)
Any anti–PD‐1 monotherapy^b^	72 (73.5)	47 (77.0)	33 (82.5)
Any anti–PD‐1 combination therapy^b^	48 (49.0)	32 (52.5)	18 (45.0)
Prior ipilimumab therapies received, No. (%)	47 (48.0)	27 (44.3)	17 (42.5)
Any ipilimumab monotherapy^b^	23 (23.5)	11 (18.0)	6 (15.0)
Any ipilimumab combination therapy^b^	26 (26.5)	19 (31.1)	12 (30.0)
Best response to prior anti–PD‐1 therapy, No. (%)			
CR	4 (4.1)	3 (4.9)	3 (7.5)
PR	11 (11.2)	9 (14.8)	4 (10.0)
SD	34 (34.7)	16 (26.2)	18 (45.0)
PD	41 (41.8)	28 (45.9)	9 (22.5)
Unknown	8 (8.2)	5 (8.2)	6 (15.0)

Abbreviations: CR, complete response; ECOG PS, Eastern Cooperative Oncology Group performance status; LDH, lactate dehydrogenase; MEK, mitogen‐activated protein kinase; PD, progressive disease; PD‐1, programmed cell death 1; PR, partial response; PS20‐A, polysorbate 20 at 0.005%–0.01%; PS20‐B, polysorbate 20 at 0.00167%; SD, stable disease.

^a^ In part 2, 31 patients began treatment with vidutolimod PS20‐A, and nine patients began treatment with vidutolimod PS20‐B.

^b^ Some patients received both monotherapy and combination therapy.

At the clinical data cutoff (August 17, 2021), four patients (4.1%) in part 1 (formulation PS20‐A) and four patients (10%) in part 2 remained on study treatment. Most patients discontinued study treatment because of PD (57.1% in part 1; 67.5% in part 2) (Table S2). Median follow‐up was 14.4 months among patients in part 1 (*n* = 98) and 5.0 months in part 2 (*n* = 40). Patient demographics and baseline characteristics were similar in those who received vidutolimod PS20‐B plus pembrolizumab (*n* = 61).

### Antitumor activity

In part 1, vidutolimod PS20‐A was evaluated in 98 patients, and PS20‐B was evaluated in 61 patients. Clinical activity data reported for part 2 combined patients initiated on vidutolimod PS20‐A (*n* = 31) and PS20‐B (*n* = 9) as monotherapy. For part 1, the antitumor activity data for vidutolimod formulation PS20‐A are presented below.

**FIGURE 1:**
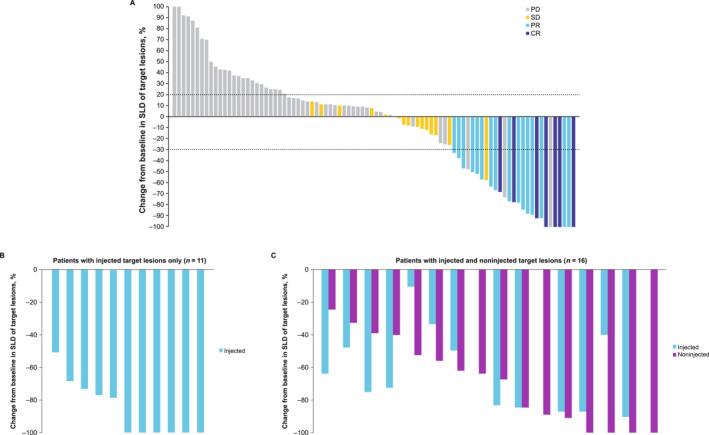
Antitumor activity in patients receiving vidutolimod PS20‐A plus pembrolizumab (*n* = 98). (A) Maximum SLD change in target lesions from baseline. (B, C) SLD change in injected and noninjected lesions in responders per RECIST v1.1 as well as postprogression responders. CR indicates complete response; PD, progressive disease; PR, partial response; PS20‐A, polysorbate 20 at 0.005%–0.01%; RECIST v1.1, Response Evaluation Criteria in Solid Tumors, version 1.1; SD, stable disease; SLD, sum of largest diameters.

In patients initiated on vidutolimod (PS20‐A) plus pembrolizumab (*n* = 98), the best ORR per RECIST v1.1 (confirmed/unconfirmed) was 23.5% (95% CI, 15.5%–33.1%), which included seven patients (7.1%) with CR and 16 patients (16.3%) with PR (Table 2). Similar response rates were observed across several baseline characteristics assessed (Figure S1). Seven patients (7.1%) achieved a 100% reduction in the sum of longest diameters of the target lesion compared with baseline (Figure 1A). Tumor volume reductions were observed in vidutolimod‐injected and noninjected target lesions (Figure 1B,C), including noninjected metastases in bone, central nervous system (CNS), liver, lung, and spleen. In patients receiving vidutolimod monotherapy (part 2), the best ORR per RECIST v1.1 was 20.0% (95% CI, 9.1%–35.6%), with all eight responders having PR (Table 2). Response rates by dose levels were also calculated for both parts (Table S3).

**TABLE 2:** Antitumor activity.

	Part 1: Vidutolimod + pembrolizumab (dose escalation and expansion)		Part 2: Vidutolimod monotherapy
	Vidutolimod PS20‐A + pembrolizumab (*N* = 98)	Vidutolimod PS20‐B + pembrolizumab (*N* = 61)	Vidutolimod (*N* = 40)
Best ORR^a^ per RECIST v1.1, % (95% CI)	23.5^b^ (15.5–33.1)	11.5^c^ (4.7–22.2)	20.0^d^ (9.1–35.6)
Best ORR^a^ including postprogression responders, % (95% CI)	27.6 (19.0–37.5)	16.4 (8.2–28.1)	22.5 (10.8–38.5)
Best response, No. (%)			
Complete response	7 (7.1)	1 (1.6)	0
Partial response	16 (16.3)	6 (9.8)	8 (20.0)
Postprogression partial response	4 (4.1)	3 (4.9)	1 (2.5)
Stable disease^e^	14 (14.3)	17 (27.9)	11 (27.5)
Progressive disease	50 (51.0)	29 (47.5)	20 (50.0)
Not evaluable	7 (7.1)	5 (8.2)	0
DOR, median (95% CI), months	25.2 (8.7–NE)	11.4 (5.4–NE)	5.6 (3.1–NE)
Time to response per RECIST v1.1, median (range), months	2.8 (2–8)	3.1 (3–11)	2.2 (1–15)
PFS per RECIST v1.1, median (95% CI), months^f^	2.9 (2.8–5.4)	2.9 (2.7–5.0)	2.1 (1.7–4.6)
6‐month PFS probability, % (95% CI)^g^	32.8 (23.5–42.4)	28.1 (17.2–39.9)	26.0 (12.6–41.7)
12‐month PFS probability, % (95% CI)	23.0 (15.0–32.0)	12.3 (5.4–22.1)	9.7 (2.5–22.9)
18‐month PFS probability, % (95% CI)	16.0 (9.3–24.3)	12.3 (5.4–22.1)	9.7 (2.5–22.9)
24‐month PFS probability, % (95% CI)	14.7 (8.3–22.9)	7.4 (2.2–16.8)	9.7 (2.5–22.9)
36‐month PFS probability, % (95% CI)	12.6 (6.4–21.0)	3.7 (0.4–14.0)	NE (NE–NE)
OS, median (95% CI), months^f^	16.3 (12.4–31.7)	25.8 (10.6–NE)	—
6‐month OS probability, % (95% CI)^g^	85.9 (76.9–91.6)	79.6 (66.8–87.9)	—
12‐month OS probability, % (95% CI)	62.4 (51.6–71.5)	63.4 (49.5–74.4)	—
18‐month OS probability, % (95% CI)	49.9 (39.2–59.8)	55.9 (42.0–67.7)	—
24‐month OS probability, % (95% CI)	44.8 (34.1–54.8)	50.1 (36.4–62.4)	—
36‐month OS probability, % (95% CI)	38.6 (27.5–49.5)	43.6 (30.0–56.4)	

Abbreviations: CI, confidence interval; DOR, duration of response; NE, not estimable; ORR, objective response rate; OS, overall survival; PFS, progression‐free survival; PS20‐A, polysorbate 20 at 0.005%–0.01%; PS20‐B, polysorbate 20 at 0.00167%; RECIST v1.1, Response Evaluation Criteria in Solid Tumors, version 1.1.

^a^ Best ORR was defined as the proportion of patients with a confirmed or unconfirmed best overall response or a complete response or partial response.

^b^ Includes 19 confirmed and four unconfirmed responses.

^c^ Includes seven confirmed and zero unconfirmed responses.

^d^ Includes four confirmed and four unconfirmed responses.

^e^ Stable disease was measured from the start of treatment until the criteria for progression were met, which took as reference the smallest sum of diameters of target lesions while on study.

^f^ Median PFS and OS and the corresponding 95% confidence intervals are calculated via Kaplan–Meier methods.

^g^ Six‐, 12‐, 18‐, 24‐, and 36‐month survival estimates and 95% confidence intervals are calculated via Kaplan–Meier methods.

**FIGURE 2:**
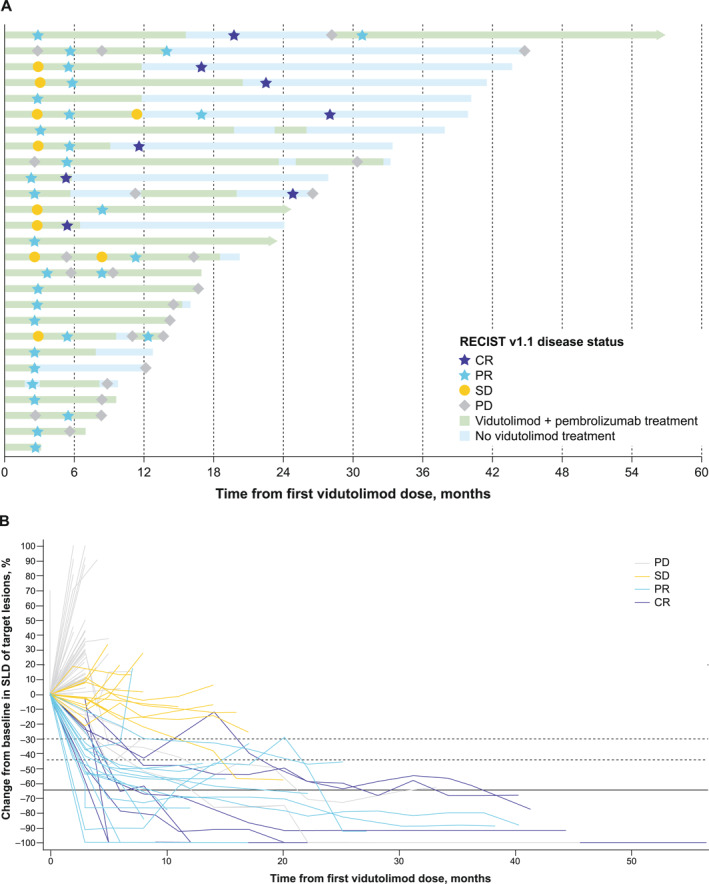
Antitumor activity over time in patients receiving vidutolimod PS20‐A plus pembrolizumab (*n* = 98). (A) Duration of follow‐up and response assessments in responders. (B) Percentage change from baseline in SLD of target lesions over time. CR indicates complete response; PD, progressive disease; PR, partial response; PS20‐A, polysorbate 20 at 0.005%–0.01%; RECIST v1.1, Response Evaluation Criteria in Solid Tumors, version 1.1; SD, stable disease; SLD, sum of largest diameters.

Median DOR was 25.2 months (95% CI, 8.7 months to not estimable [NE]) with vidutolimod (PS20‐A) plus pembrolizumab (Table 2). Twelve patients had a response for >12 months, and two of these patients had a response for >36 months (Figure 2). In patients receiving vidutolimod monotherapy, median DOR was 5.6 months (95% CI, 3.1 months to NE) (Table 2). Median PFS with vidutolimod (PS20‐A) plus pembrolizumab was 2.9 months (95% CI, 2.8–5.4 months), and 2.1 months (95% CI, 1.7–4.6 months) with vidutolimod monotherapy. The probability of PFS was 32.8% and 23.0% at 6 and 12 months, respectively, for patients who received vidutolimod (PS20‐A) plus pembrolizumab (Table 2). Median OS with vidutolimod (PS20‐A) plus pembrolizumab was 16.3 months (95% CI, 12.4–31.7 months). The probability of OS was 85.9% and 62.4% at 6 and 12 months, respectively, for these patients (Table 2). OS data were not collected in part 2 per the statistical analysis plan. Clinical activity in patients who received vidutolimod PS20‐B plus pembrolizumab is presented in Table 2. This formulation is no longer in clinical development.

### Safety

In part 1, patients had a median of eight vidutolimod injection visits (range, 1–62), with a median of one injection per visit (range, 1–3.7), and received a median of four pembrolizumab doses (range, 1–58). In part 2, patients had a median of 11.5 vidutolimod injection visits (range, 3–45), with a median of one injection per visit (range, 1–6).

Any‐grade treatment‐emergent adverse events (TEAEs) were reported in 159 patients (100.0%) in part 1, and 40 patients (100.0%) in part 2 (Table 3). The safety profile was similar in patients receiving vidutolimod plus pembrolizumab versus vidutolimod monotherapy. Grade ≥3 TEAEs were reported in 88 patients (55.3%) in part 1, and 15 patients (37.5%) in part 2 (Table 3). No grade 4 TEAEs were reported in part 2. The most common any‐grade TEAEs in parts 1 and 2 included chills (76.7% and 60.0%, respectively), pyrexia (62.9% and 50.0%), fatigue (57.9% and 37.5%), and nausea (56.0% and 57.5%) (Table 4). There were no treatment‐related deaths in either part. During the part 1 dose‐escalation and dose‐expansion phases, three patients (1.9%) had a TEAE resulting in death—one patient with a past history of infected neoplasm died of sepsis, and two patients with baseline lung metastases died of respiratory failure after experiencing worsening of lung metastases on study. There were no deaths due to TEAEs in part 2. The most common grade ≥3 TEAEs (reported in >5% of patients) in parts 1 and 2 included hypotension (8.2%, *n* = 13 and 5%, *n* = 2, respectively) and hypertension (6.9%, *n* = 11 and 15%, *n* = 6, respectively).

**TABLE 3:** Safety summary.

	Part 1: Vidutolimod + pembrolizumab (dose escalation and expansion)			Part 2: Vidutolimod monotherapy
	Vidutolimod + pembrolizumab (*N* = 159)	Vidutolimod PS20‐A + pembrolizumab (*N* = 98)	Vidutolimod PS20‐B + pembrolizumab (*N* = 61)	Vidutolimod^a^ (*N* = 40)
Patients with ≥1 any‐grade TEAEs, No. (%)^b^	159 (100.0)	98 (100.0)	61 (100.0)	40 (100.0)
Grade 3^c^	73 (45.9)	48 (49.0)	25 (41.0)	15 (37.5)
Grade 4^c^	12 (7.5)	7 (7.1)	5 (8.2)	0
Grade 5^c^	3 (1.9)	1 (1.0)	2 (3.3)	0
Serious TRAE, No. (%)	26 (16.4)	17 (17.3)	9 (14.8)	6 (15.0)
TEAEs leading to discontinuation of vidutolimod, No. (%)	15 (9.4)	8 (8.2)	7 (11.5)	3 (7.5)

Abbreviations: PS20‐A, polysorbate 20 at 0.005%–0.01%; PS20‐B, polysorbate 20 at 0.00167%; TEAE, treatment‐emergent adverse event; TRAE, treatment‐related adverse event.

^a^ Includes vidutolimod PS20‐A and vidutolimod PS20‐B.

^b^ TEAEs were graded via National Cancer Institute Common Terminology Criteria for Adverse Events, version 5.0.

^c^ Patients are reported on the basis of the highest severity TEAE.

**TABLE 4:** Adverse events.^a^ ^,^ ^b^

	Part 1: Vidutolimod + pembrolizumab (dose escalation and expansion) (*N* = 159)^a^			Part 2: Vidutolimod monotherapy (*N* = 40)^b^		
	Any‐grade TEAEs, No. (%)	Grade 3 TEAEs, No. (%)	Grade 4 TEAEs, No. (%)	Any‐grade TEAEs, No. (%)	Grade 3 TEAEs, No. (%)	Grade 4 TEAEs, No. (%)
Chills	122 (76.7)	5 (3.1)	—	24 (60.0)	—	—
Pyrexia	100 (62.9)	4 (2.5)	—	20 (50.0)	1 (2.5)	—
Fatigue	92 (57.9)	4 (2.5)	—	15 (37.5)	0	—
Nausea	89 (56.0)	—	—	23 (57.5)	—	—
Vomiting	66 (41.5)	—	—	10 (25.0)	—	—
Injection site pain	50 (31.4)	1 (0.6)	—	6 (15.0)	—	—
Headache	54 (34.0)	1 (0.6)	—	17 (42.5)	1 (2.5)	—
Back pain	45 (28.3)	6 (3.8)	—	10 (25.0)	1 (2.5)	—
Hypotension	40 (25.2)	11 (6.9)	2 (1.3)	11 (27.5)	2 (5.0)	—
Arthralgia	31 (19.5)	3 (1.9)	—	6 (15.0)	—	—
Pruritus	21 (13.2)	1 (0.6)	—	12 (30.0)	—	—
Hypertension	22 (13.8)	11 (6.9)	—	9 (22.5)	6 (15.0)	—
AST increased	17 (10.7)	4 (2.5)	1 (0.6)	2 (5.0)	1 (2.5)	—
ALT increased	14 (8.8)	2 (1.3)	1 (0.6)	3 (7.5)	1 (2.5)	—
Anemia	22 (13.8)	7 (4.4)	—	6 (15.0)	2 (5.0)	—
Hypoxia	10 (6.3)	6 (3.8)	—	2 (5.0)	—	—
Hypophosphatemia	9 (5.7)	5 (3.1)	—	3 (7.5)	—	—
Sepsis	5 (3.1)^c^	1 (0.6)	3 (1.9)	1 (2.5)	—	—
Cellulitis	10 (6.3)	4 (2.5)	—	2 (5.0)	—	—
Hyponatremia	11 (6.9)	5 (3.1)	—	—	—	—
Hypokalemia	15 (9.4)	3 (1.9)	—	2 (5.0)	0	—
Muscular weakness	10 (6.3)	3 (1.9)	—	3 (7.5)	1 (2.5)	—
Pain in extremity	24 (15.1)	3 (1.9)	—	3 (7.5)	—	—
Encephalopathy	3 (1.9)	3 (1.9)	—	—	—	—
Diarrhea	49 (30.8)	3 (1.9)	—	10 (25.0)	0	—

Abbreviations: ALT, alanine aminotransferase; AST, aspartate aminotransferase; TEAE, treatment‐emergent adverse event.

^a^ Any‐grade TEAEs reported in >25% of patients and grade 3/4 TEAEs reported in ≥3 patients in part 1 or part 2. In part 1, adverse events (grade ≥3) included one patient (0.6%) with pneumonitis and one patient (0.6%) with adrenal insufficiency.

^b^ TEAEs were graded via National Cancer Institute Common Terminology Criteria for Adverse Events, version 5.0.

^c^ Treatment‐emergent grade 5 sepsis was reported in one patient.

## DISCUSSION

For PD‐1 blockade–resistant melanoma, new immunotherapy combinations are needed to simultaneously target multiple cancer immune evasion mechanisms.^20^, ^21^ This phase 1b study evaluated the safety and clinical activity of vidutolimod with or without pembrolizumab in heavily pretreated patients.

Consistent with the results previously reported for the dose‐escalation part,^16^ the final analysis of the study with additional follow‐up, including more patients with PD‐1 blockade–resistant melanoma, showed antitumor activity of vidutolimod as a single agent, as well as in combination with pembrolizumab. Intratumoral vidutolimod PS20‐A (the formulation selected for further clinical development because of decreased antitumor activity of vidutolimod PS20‐B) plus iv pembrolizumab demonstrated promising antitumor activity with a robust ORR of 23.5%. Notably, this study’s patient population with anti–PD‐1–resistant melanoma included patients who had received prior ipilimumab. The response rate with vidutolimod PS20‐A plus pembrolizumab was similar to those observed in studies investigating second‐line treatment with ipilimumab plus pembrolizumab or nivolumab.^22^, ^23^, ^24^ Tumor regression in response to vidutolimod plus pembrolizumab was observed in both injected and distant noninjected lesions, including bone, CNS, and visceral metastases. These findings support the premise that local activation of tumor‐associated pDCs by intratumoral vidutolimod can initiate a systemic antitumor response.

To our knowledge, vidutolimod is the only TLR9 agonist to demonstrate systemic single‐agent antitumor activity in advanced cutaneous melanoma, with a RECIST v1.1 ORR of 20.0%. SD‐101, a CpG‐C TLR9 agonist, had transient single‐agent activity in murine models that has not been confirmed in patients with advanced nonocular melanoma.^25^, ^26^ Tilsotolimod, a CpG‐C TLR9 agonist, appeared to have promising clinical activity when combined with ipilimumab in early‐phase studies^27^; however, no RECIST v1.1 responses were observed with tilsotolimod monotherapy (*n* = 54, including 16 patients with melanoma).^28^ Furthermore, in a phase 3 study, tilsotolimod plus ipilimumab did not improve ORR or OS versus ipilimumab alone in anti–PD‐1–resistant advanced melanoma.^29^ Because monotherapy activity has been identified as a key predictor of clinical benefit for combination treatments,^30^ the monotherapy activity of vidutolimod demonstrated in this study may be the most important evidence of differentiation from other agents evaluated as immunotherapy combination partners in PD‐1 blockade–resistant, advanced nonocular melanoma.

Other therapeutic approaches are also being explored in patients with PD‐1 blockade–resistant melanoma. Tumor‐derived T‐cell immunotherapy, a cellular therapy, was recently approved by the US Food and Drug Administration in patients with unresectable or metastatic melanoma who have previously received other therapies such as anti–PD‐1 therapy, and in patients with tumors that are *BRAF* V600 mutation positive, a BRAF inhibitor with or without a mitogen‐activated protein kinase (MEK) inhibitor.^31^ Further clinical development of vidutolimod may also be attractive in earlier stages of melanoma, given that the combination of neoadjuvant vidutolimod plus anti–PD‐1 therapy demonstrated encouraging pathologic response rates in a single‐arm pilot study for patients with resectable stage III melanoma,^32^ which led to a randomized phase 2 study (ClinicalTrials.gov identifier NCT04708418).

In our study, ORRs were comparable between the vidutolimod monotherapy and vidutolimod PS20‐A plus pembrolizumab arms (20.0% and 23.5%, respectively). With the caveat that there were imbalances between the groups regarding sites of disease at baseline, it is notable that vidutolimod demonstrated single‐agent antitumor activity in advanced cutaneous melanoma. Median DOR was 25.2 months on treatment with vidutolimod (PS20‐A) plus pembrolizumab, substantially longer than for vidutolimod monotherapy, which supports the hypothesis that combination with an anti–PD‐1 agent is critical to counteract the TLR9‐mediated upregulation of PD‐L1 and allow for sustained activity of antitumor CD8+ T cells activated by the TLR9 agonist. This may also explain why tilsotolimod in combination with ipilimumab (anti–CTLA‐4) did not improve ORR or OS in a phase 3 trial in advanced PD‐1 blockade–resistant melanoma.^29^

Vidutolimod with or without pembrolizumab demonstrated an acceptable safety profile. The most common AEs were transient injection site reactions and flu‐like symptoms, which are expected on the basis of the mechanism of action of vidutolimod. Vidutolimod plus pembrolizumab demonstrated tolerability in patients with PD‐1 blockade–resistant melanoma, as supported by the incidence of grade ≥3 AEs and severe immune‐mediated AEs and the absence of treatment‐related deaths. Despite frequent TEAEs (including grades 3 and 4), the frequency of serious TRAEs was 16.4% in part 1 and 15.0% in part 2.

Study limitations were those typical for a phase 1 clinical trial, including small sample size, lack of a control group, and limited duration of follow‐up.

In conclusion, the durable, systemic antitumor activity and acceptable safety profile demonstrated in this phase 1b study support further clinical development of vidutolimod in combination with PD‐1 blockade to target cancer immune evasion and overcome PD‐1 blockade resistance.

## AUTHOR CONTRIBUTIONS

**Mohammed M. Milhem**: Conceptualization, investigation, supervision, writing–original draft, and writing–review and editing. **Yousef Zakharia**: Writing–review and editing. **Diwakar Davar**: Writing–original draft and writing–review and editing. **Elizabeth I. Buchbinder**: Investigation and writing–review and editing. **Theresa Medina**: Writing–review and editing. **Adil Daud**: Writing–review and editing. **Antoni Ribas**: Data curation, formal analysis, investigation, resources, supervision, and writing–review and editing. **Bartosz Chmielowski**: Data curation, formal analysis, investigation, project administration, and writing–review and editing. **Jiaxin Niu**: Investigation, supervision, and writing–review and editing. **Geoffrey T. Gibney**: Writing–review and editing. **Kim Margolin**: Writing–review and editing. **Anthony J. Olszanski**: Data curation, investigation, supervision, and writing–review and editing. **Inderjit Mehmi**: Data curation, investigation, and writing–review and editing. **Takami Sato**: Writing–review and editing. **Montaser Shaheen**: Writing–review and editing. **Luping Zhao**: Writing–review and editing. **Heather Kelley**: Writing–review and editing. **Hong Liu**: Writing–review and editing. **Sujatha Kumar**: Writing–review and editing. **Dmitri Bobilev**: Writing–review and editing. **Arthur M. Krieg**: Conceptualization, funding acquisition, investigation, methodology, project administration, resources, supervision, writing–original draft, and writing–review and editing. **James E. Wooldridge**: Data curation, formal analysis, funding acquisition, project administration, resources, visualization, and writing–review and editing. **John M. Kirkwood**: Investigation, writing–review and editing.

## CONFLICT OF INTEREST STATEMENT

Yousef Zakharia reports being a member of advisory boards for Bristol‐Myers Squibb, Eisai, EMD Serono, Exelixis, Gilead, Pfizer, and Seagen, and being a member of a steering committee, data and safety monitoring committee, or adjudication panel for Janssen. Diwakar Davar reports grants or research support to the institution from Arcus Biosciences, Immunocore, Merck, Regeneron Pharmaceuticals, and Tesaro/GlaxoSmithKline; serving as a consultant and receiving compensation from ACM Biolabs, Ascendis, Castle Biosciences, Clinical Care Options, Gerson Lehrman Group (GLG), Immunitas Therapeutics, Med Learning Group, Replimune, TriSalus Life Sciences, and Xilio Therapeutics; participating in a CE speakers’ bureau for Castle Biosciences; being a member of a steering committee or data and safety monitoring board for Replimune and Immunocore; stock ownership in mBiomics and Zola Therapeutics; being a member of advisory boards for ACM Bio; and two patent applications: US Patent 63/124,231, “Compositions and Methods for Treating Cancer,” December 11, 2020, and US Patent 63/208,719, “Compositions and Methods for Responsiveness to Immune Checkpoint Inhibitors (ICI), Increasing Effectiveness of ICI and Treating Cancer,” June 9, 2021. Elizabeth I. Buchbinder reports serving as a consultant for or being a member of advisory boards for Pfizer, Werewolf Therapeutics, Merck, Nektar, Obsidian Therapeutics, Instil Bio, Bristol‐Myers Squibb, Zola Therapeutics, ANAVEON, Iovance Biotherapeutics, Sanofi, Xilio Therapeutics, and Novartis Pharmaceuticals, and receiving clinical trial support from Lilly, Novartis Pharmaceuticals, Partners Therapeutics, Genentech, and BVD. Theresa Medina reports serving as a consultant for Mural Oncology, Moderna, Iovance Biotherapeutics, Pfizer, Regeneron Pharmaceuticals, Merck, Genentech, Replimune, and Bristol‐Myers Squibb; research trial support to the institution from Bristol‐Myers Squibb, Genentech, Iovance Biotherapeutics, Merck, Regeneron Pharmaceuticals, Replimune, and Pfizer; and travel support from Regeneron Pharmaceuticals. Adil Daud reports honoraria from EMD Serono and Inovio Pharmaceuticals; a consulting or advisory role for Genoptix, GlaxoSmithKline, Merck, Pfizer, Bristol‐Myers Squibb, OncoSec, Caris Life Sciences, Eisai, and GLG; research funding from Bristol‐Myers Squibb, Checkmate Pharmaceuticals, Incyte, and Novartis Pharmaceuticals; research funding to the institution from Genentech/Roche, GlaxoSmithKline, Merck/Schering‐Plough, OncoSec, and Pfizer; patents or royalties from or other intellectual property with TREX and OncoSec; and stock and other ownership interests in NEUVOGEN and TRexBio. Antoni Ribas reports honoraria from consulting for Amgen, Apricity Health, Arsenal Bio, Compugen, Genentech/Roche, Lyell Immunopharma, MapKure, Merus, Synthekine, and Tango; being a current or former member of scientific advisory boards; having stock in Appia Bio, Apricity Health, Arcus Biosciences, Compugen, CytomX Therapeutics, Highlight Therapeutics, ImaginAb, ImmPact, Inspirna, Kite/Gilead, Larkspur, Lutris Pharma, Lyell Immunopharma, MapKure, Merus, Synthekine, and Tango; research funding from Agilent and Bristol‐Myers Squibb via Stand Up to Cancer (SU2C); serving as a fiduciary officer for Lutris Pharma and Arcus Biosciences; and patent royalties from Arsenal Bio. Bartosz Chmielowski reports being a member of advisory boards for Atreca, Fortvita Biologics, Immunocore, Regeneron Pharmaceuticals, and Treeline Biosciences; being a member of data monitoring committees for Servier, SpringWorks Therapeutics, and TuHURA; and research trial support from Adagene, Advenchen Laboratories, Ascentage, AskGene Pharma, Atreca, Bristol‐Myers Squibb, Compugen, Georgiamune, IDEAYA Biosciences, Immatics, Immunocore, Infinity Pharmaceuticals, InstilBio, Iovance Biotherapeutics, Kezar Life Sciences, Kinnate, Krystal Biotech, Macrogenics, Nested Therapeutics, Pierre Fabre, PTC Therapeutics, RAPT Therapeutics, Replimune, TriSalus Life Sciences, Xencor, and Xilio Therapeutics. Jiaxin Niu reports a consulting or advisory role for AstraZeneca, Bristol‐Myers Squibb, Daiichi Sankyo, EMD Serono, G1 Therapeutics, Johnson & Johnson, Merck, Pfizer, Sanofi, and Takeda. Geoffrey T. Gibney reports being a consultant or on an advisory board for Bristol‐Myers Squibb, Immunocore, Incyte, Iovance Biotherapeutics, Lyell Immunopharma, Merck, Novartis Pharmaceuticals, Pfizer, and Replimune; being a member of a data and safety monitoring board for HUYABIO; and research support from Exelixis. Kim Margolin reports being a member of a data and safety monitoring board for Elicio and Iovance Biotherapeutics; being a member of the adjuvant trial steering committee and a local trial principal investigator at Regeneron Pharmaceuticals; research trial support from Agenus and Immunocore; being a member of an advisory board for T‐NanoBio; serving as a consultant for BeiGene, AstraZeneca, Bristol‐Myers Squibb, Daiichi Sankyo, ImaginAb, Merck, Tallac, and Werewolf Therapeutics; and being a global trial principal investigator at ImaginAb. Anthony J. Olszanski reports advisory board consulting fees from IO Biotech, Merck, Natera, Replimune, OncoSec, Pfizer, ANAVEON, and Bristol‐Myers Squibb; being a member of data and safety monitoring boards for Pfizer and Takeda; and research support to the institution from ANAVEON, Antegene, Clasp, GlaxoSmithKline, HiFiBiO, Immatics, Janssen, Lantern, Perspectives, Pfizer, Regeneron Pharmaceuticals, Replimune, Shionogi, and Takeda. Inderjit Mehmi reports participating in speakers’ bureaus for Bristol‐Myers Squibb and Immunocore; serving as a consultant for Guidepoint and GLG; and stock and other ownership interests in Immunocore, IDEAYA Biosciences, Iovance Biotherapeutics, and Delcath. Takami Sato reports serving as a consultant or a member of advisory boards for Castle Biosciences and Immunocore, and research trial support to the institution from Immunocore. Luping Zhao reports holding stock with Sarepta Therapeutics. Dmitri Bobilev reports former employment with Checkmate Pharmaceuticals. Arthur M. Krieg reports being the founder and CEO of Zola Therapeutics; holding stock with Zola Therapeutics; being a trial sponsor and having a previous affiliation with Checkmate Pharmaceuticals; and being a consultant for PepGen and Strand Therapeutics. James E. Wooldridge reports a previous affiliation with Checkmate Pharmaceuticals and current affiliation with Immunitas Therapeutics, and being a consultant for Regeneron Pharmaceuticals. John M. Kirkwood reports being a consultant or a member of scientific advisory boards for Ankyra Therapeutics, Axio Research, Boxer Capital, Bristol‐Myers Squibb, CytomX Therapeutics, Daiichi Sankyo, Engage Health Media, iOnctura, Iovance Biotherapeutics, IQVIA, Istari Oncology, Jazz Pharmaceuticals, Lumira Capital Investment Management, Lytix Biopharma, Magnolia Innovation, Merck, Mural Oncology, Natera, Novartis Pharmaceuticals, OncoCyte, PathAI, Pfizer, Piper Sandler, Pyrojas, Regeneron Pharmaceuticals, Replimune, Scopus BioPharma, Takeda, Valar Labs, and Zola Therapeutics; and research trial support to the institution from Amgen, Bristol‐Myers Squibb, Checkmate Pharmaceuticals, Harbour BioMed, Immunocore, ImmVira Pharma, Iovance Biotherapeutics, Lytix Biopharma, Novartis Pharmaceuticals, Takeda, and Verastem. The other authors declare no conflicts of interest.

## Supporting information

Supplementary Material

## Data Availability

Qualified researchers may request access to study documents (including the clinical study report, study protocol with any amendments, blank case report form, and statistical analysis plan) that support the methods and findings reported in this article. Individual anonymized participant data will be considered for sharing once the product and indication have been approved by major health authorities (e.g., Food and Drug Administration, European Medicines Agency, Pharmaceuticals and Medical Devices Agency, etc.) if there is legal authority to share the data and there is not a reasonable likelihood of participant reidentification. Requests should be submitted to https://vivli.org/.
