# Supplementary material for: Intratumoral vidutolimod as monotherapy or in combination with pembrolizumab in patients with programmed cell death 1 blockade–resistant melanoma: Final analysis from a phase 1b study
Source: Cancer. 2025 Aug 3;131(15):e70022. doi: 10.1002/cncr.70022 (PMC12318270; doi:10.1002/cncr.70022)
Supplement: Supplementary file 1 — Supplementary Material [file CNCR-131-e70022-s001.docx]

**SUPPORTING INFORMATION**

**Supplementary Section A1. Additional eligibility criteria**

PD-1 blockade was not required to be the most recent treatment prior to study entry. Key exclusion criteria were anti–cytotoxic T-lymphocyte associated protein 4 or investigational therapy administered within 30 days, need for systemic oral corticosteroids of >10 mg/day (prednisone equivalent) at the time of enrollment, history of grade 4 immune-mediated toxicity, and known active central nervous system metastases.

**Supplementary Section A2. Guidance on vidutolimod injections and tumor selection**

Vidutolimod was recommended to be injected into the most aggressively growing accessible lesions according to the judgment of the investigator. Visceral tumors could have been injected if, in the opinion of the investigator, they were deemed the most appropriate tumor for intratumoral injection. The total volume of vidutolimod dose could be split among several lesions at the investigator’s discretion. The protocol stipulated that only the minimum number of lesions that could be injected by the ordered volume was to be chosen. If a new lesion appeared or if the injected lesion regressed, another accessible lesion could be injected. If no accessible lesions remained, vidutolimod could be injected subcutaneously in the area of former disease.

**Supplementary Table S1.** List of institutional review boards or independent ethics committees.

|  | IRB/IEC (Name and Address) | Sites |
| --- | --- | --- |
| 1 | WCG IRB  1019 39th Ave., SE, Suite 120  Puyallup, WA 98374  (central IRB for study) | 1. Gabrail Cancer Center 2. University of Iowa 3. University of Pittsburgh Medical Center 4. University of Colorado 5. Fox Chase 6. University of Arizona 7. Banner MD Anderson 8. Thomas Jefferson University |
| 2 | UCLA IRB  10889 Wilshire Blvd, Suite 830  Los Angeles, CA 90095-1406 | 1. University of California, Los Angeles |
| 3 | DFCI Office for Human Research Studies  450 Brookline Ave  Boston, MA 02215-5450 | 1. Dana-Farber Cancer Institute 2. Massachusetts General Hospital 3. Beth Israel Deaconess Medical Center |
| 4 | NYU School of Medicine IRB,  1 Park Ave, 6th Floor,  New York, NY 10016 | 1. NYU |

|  | IRB/IEC (Name and Address) | Sites |
| --- | --- | --- |
| 5 | West Virginia University IRB,  886 Chestnut Ridge Rd, Morgantown,  WV 26506 | 1. West VA |
| 6 | City of Hope IRB,  1500 East Duarte Road,  Duarte, CA 91010-3000 | 1. City of Hope |
| 7 | Georgetown University IRB,  Medical-Dental Building SW104,  3900 Reservoir Road NW  Washington, DC 20057 | 1. Georgetown |
| 8 | UCSF HRRP/IRB,  490 Illinois St, Floor 6,  San Francisco, CA 94158 | 1. University of California, San Francisco |

Abbreviations: IRB, institutional review board; IEC, independent ethics committee.

**Supplementary Table S2.** Patient disposition.

|  | Part 1: Vidutolimod + Pembrolizumab  (Dose Escalation and Expansion) | | Part 2: Vidutolimod Monotherapy |
| --- | --- | --- | --- |
|  | **Vidutolimod PS20-A + Pembrolizumab**  **(*N* = 98)** | **Vidutolimod**  **PS20-B + Pembrolizumab**  **(*N* = 61)** | **Vidutolimod^a^**  **(*N* = 40)** |
| Treated patients, n (%) | 98 (100) | 61 (100) | 40 (100) |
| Enrolled into treatment extension^b^ | 4 (4.1) | 4 (6.6) | 4 (10.0) |
| Discontinued study treatment | 94 (95.9) | 57 (93.4) | 36 (90.0) |
| PD per RECIST v1.1 accompanied by medically significant deterioration | 56 (57.1) | 37 (60.7) | 27 (67.5) |
| Withdrew consent | 15 (15.3) | 6 (9.8) | 3 (7.5) |

|  | Part 1: Vidutolimod + Pembrolizumab  (Dose Escalation and Expansion) | | Part 2: Vidutolimod Monotherapy |
| --- | --- | --- | --- |
|  | **Vidutolimod PS20-A + Pembrolizumab**  **(*N* = 98)** | **Vidutolimod PS20-B + Pembrolizumab**  **(*N* = 61)** | **Vidutolimod^a^**  **(*N* = 40)** |
| AE | 4 (4.1) | 5 (8.2) | 3 (7.5) |
| Death | 0 | 2 (3.3) | 0 |
| Investigator decision | 9 (9.2) | 2 (3.3) | 2 (5.0) |
| Dose-limiting or other unacceptable toxicity considered related to study medication | 1 (1.0) | 0 | 0 |
| Other | 9 (9.2) | 5 (8.2) | 1 (2.5) |

Abbreviations: AE, adverse event; PD, progressive disease; PS20-A, polysorbate 20 at 0.005%–0.01%; PS20-B, polysorbate 20 at 0.00167%; RECIST v1.1, Response Evaluation Criteria in Solid Tumors, version 1.1.

^a^Includes vidutolimod PS20-A and vidutolimod PS20-B.

^b^Ongoing at the time of clinical data cutoff.

**Supplementary Table S3.** Response rates by dose level.

|  | Part 1: Vidutolimod PS20-A + Pembrolizumab  (Dose Escalation and Expansion)  (*N* = 98) | | | | | Part 2: Vidutolimod Monotherapy^a^  (*N* = 40) | |  |
| --- | --- | --- | --- | --- | --- | --- | --- | --- |
| Dose | **1 mg**  **(*n* = 3)** | **3 mg**  **(*n* = 16)** | **5 mg**  **(*n* = 9)** | **7.5 mg**  **(*n* = 6)** | **10 mg**  **(*n* = 64)** | **5 mg**  **(*n* = 15)** | **10 mg**  **(*n* = 25)** | |
| Best ORR per RECIST v1.1, % (95% CI) | 0.0  (0.0–70.8) | 18.8  (4.0–45.6) | 44.4  (13.7–78.8) | 0.0  (0.0–45.9) | 25.0  (15.0–37.4) | 20.0  (4.3–48.1) | 20.0  (6.8–40.7) | |
| Best ORR including post-progression responders, % (95% CI) | 0.0  (0.0–70.8) | 18.8  (4.0–45.6) | 44.4  (13.7–78.8) | 16.7  (0.4–64.1) | 29.7  (18.9–42.4) | 20.0  (4.3–48.1) | 24.0  (9.4–45.1) | |
| Best response, n (%) |  |  |  |  |  |  |  | |
| Complete response | 0 | 1 (6.3) | 2 (22.2) | 0 | 4 (6.3) | 0 | 0 | |
| Partial response | 0 | 2 (12.5) | 2 (22.2) | 0 | 12 (18.8) | 3 (20.0) | 5 (20.0) | |
| Post-progression partial response | 0 | 0 | 0 | 1 (16.7) | 3 (4.7) | 0 | 1 (4.0) | |
| Stable disease | 0 | 1 (6.3) | 1 (11.1) | 2 (33.3) | 11 (17.2) | 3 (20.0) | 9 (36.0) | |
| Progressive disease | 3 (100.0) | 10 (62.5) | 3 (33.3) | 4 (66.7) | 33 (51.6) | 9 (60.0) | 11 (44.0) | |
| Not evaluable | 0 | 2 (12.5) | 1 (11.1) | 0 | 4 (6.3) | 0 | 0 | |

Abbreviations: CI, confidence interval; ORR, objective response rate; PS20-A, polysorbate 20 at 0.005%–0.01%; RECIST v1.1, Response Evaluation Criteria in Solid Tumors version 1.1.

^a^Includes vidutolimod PS20-A and vidutolimod PS20-B.

**Supplementary Figure S1.** Antitumor activity in patients receiving vidutolimod PS20-A + pembrolizumab (*n* = 98). Forest plot of objective response rates in patient populations defined by baseline characteristics.


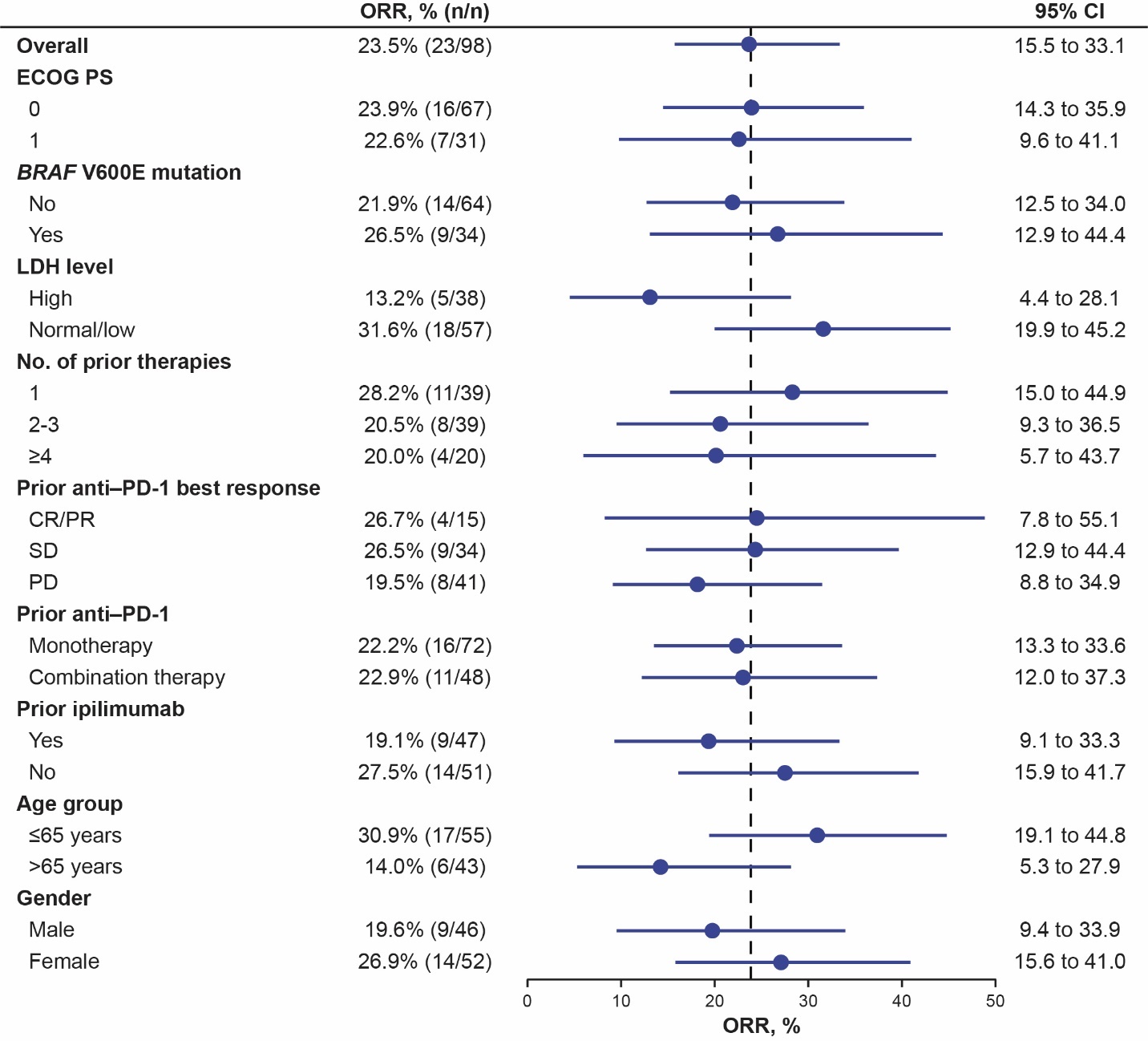


Abbreviations: CI, confidence interval; CR, complete response; ECOG PS, Eastern Cooperative Oncology Group performance status; LDH, lactate dehydrogenase; ORR, objective response rate; PD, progressive disease; PD-1, programmed cell death protein-1; PR, partial response; SD, stable disease.
